# Overview of the Anticancer Profile of Avenanthramides from Oat

**DOI:** 10.3390/ijms20184536

**Published:** 2019-09-13

**Authors:** Eleonora Turrini, Francesca Maffei, Andrea Milelli, Cinzia Calcabrini, Carmela Fimognari

**Affiliations:** Department for Life Quality Studies, Alma Mater Studiorum-Università di Bologna, Corso d’Augusto 237, 47921 Rimini, Italy; eleonora.turrini@unibo.it (E.T.); francesca.maffei@unibo.it (F.M.); andrea.milelli3@unibo.it (A.M.); cinzia.calcabrini@unibo.it (C.C.)

**Keywords:** oat, avenanthramides, cancer therapy, cancer chemoprevention, anticancer mechanisms, pharmacokinetics, toxicological profile

## Abstract

Cancer represents one of the leading causes of death worldwide. Progresses in treatment of cancer have continued at a rapid pace. However, undesirable side effects and drug resistance remain major challenges for therapeutic success. Natural products represent a valuable starting point to develop new anticancer strategies. Polyphenols, well-known as antioxidant, exert anticancer effects through the modulation of multiple pathways and mechanisms. Oat (*Avena sativa* L., *Poaceae*) is a unique source of avenanthramides (AVAs), a group of polyphenolic alkaloids, considered as its signature compounds. The present review aims to offer a comprehensive and critical perspective on the chemopreventive and chemotherapeutic potential of AVAs. AVAs prevent cancer mainly by blocking reactive species. Moreover, they exhibit potential therapeutic activity through the modulation of different pathways including the activation of apoptosis and senescence, the block of cell proliferation, and the inhibition of epithelial mesenchymal transition and metastatization. AVAs are promising chemopreventive and anticancer phytochemicals, which need further clinical trials and toxicological studies to define their efficacy in preventing and reducing the burden of cancer diseases.

## 1. Introduction

Fiber-rich and unrefined food has been found to decrease the risk of several chronic diseases, including cancer [1,2]. Cancer is the second cause of mortality all over the world [3]. Although the availability of many chemotherapeutic agents, toxicity and chemoresistance are common problems compromising their clinical effectiveness [4]. Natural products represent a valuable starting point to develop new anticancer strategies [5,6,7]. The ability of natural products to synergistically interact with multiple cellular targets can help counteracting the complexity of cancer pathogenesis. Moreover, natural compounds are usually characterized by a better safety profile, compared to traditional chemotherapeutic drugs [8].

Oat (*Avena sativa* L., *Poaceae*) is an annual 1.5-m-high grass widely cultivated in cool and moist region of Northern Europe and North America [9]. After removal of the inedible outer hull, the remaining oat groats are processed to obtain oatmeal, naturally containing oat bran. Oat is mainly eaten as porridge, breakfast cereals, and baked goods (oatcakes, oat cookies, and oat bread). Initially, the interest in whole grain oat was mainly due to its advantageous macronutrient composition including lipids with a high degree of unsaturation and fibers with high content of β-glucans [10]. Then, scientific evidence has shown that oat contains other important bioactive compounds, such as polyphenols, which share with fibers a protective effect against the development of chronic degenerative diseases, including cancer [11]. In particular, besides phenolic acids and flavonoids, oat is a unique source of avenanthramides (AVAs), a group of phenolic alkaloids considered its signature compounds. AVAs were firstly isolated by Collins and colleagues [12] and represent the major alcohol-soluble phenolic antioxidants in oat. In the plant, they act as key phytoalexins against plant pathogens. AVAs are constitutively expressed in the hulls, groats, bran, and outer layers of the oat kernel [12,13].

AVAs modulate multiple biological events, resulting in anti-inflammatory, anti-itching, and immunomodulatory effects [14]. Different studies indicate that AVAs also exert antioxidant and antiproliferative effects, which help preventing or treating cancer [10,15].

The aim of the present review is to provide a critical overview of the cancer chemopreventive and chemotherapeutic activity of AVAs, as single molecules or oat preparations. Emphasis is placed on the mechanisms triggered by these phytochemicals to counteract cancer pathogenesis. Besides, the review highlights the epidemiological studies investigating the preventive role of oat dietary intake on cancer risk.

## 2. Chemical Structure of AVAs and Their Derivatives

**Figure 1:**
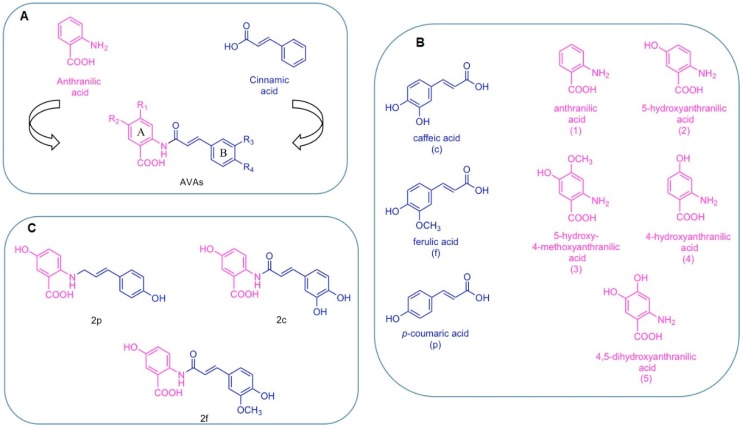
(**A**) General structure of avenanthramides (AVAs). (**B**) Derivatives of cinnamic and anthranilic acids find in AVAs and respective nomenclature. (**C**) Most abundant AVAs.

Structurally, AVAs are substituted *N*-cinnamoyl anthranilic acids composed of the combination of two fragments: the left side of the molecule derives from anthranilic acid and the right side derives from cinnamic acid (Figure 1A). Based on this particular structure and on the substitution patterns, a systematic nomenclature has been developed associating letters and numbers to each substituent [16,17]. The three main cinnamic acid derivatives are caffeic acid, indicated with the letter (c), ferulic acid (f), and *p*-coumaric acid (p) (Figure 1B). The five differently substituted anthranilic acids are anthranilic acid, indicated as (1), 5-hydroxyanthranilic acid (2), 5-hydroxy-4-methoxyanthranic acid (3), 4-hydroxyanthranic acid (4), 4,5-dihydroxyanthranic acid (5) (Figure 1B).

Among the 25 AVAs congeners found in oat, 2c, 2f and 2p (Figure 1C) are the most abundant and better characterized for their biological profiles.

**Figure 2:**
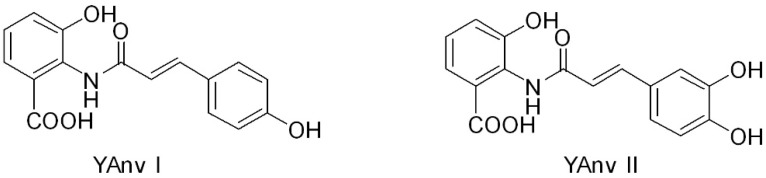
Structures of yeast-derived AVAs.

Considering the wide range of potential therapeutic applications of AVAs, genetic engineering strategies as alternative to conventional chemical synthesis were developed to help AVAs production on an industrial scale. [18]. For instance, AVAs can be produced through genetically engineered microorganisms, including *Saccharomyces cerevisiae* and *Escherichia coli*. Two novel yeast-derived recombinant AVAs [i.e., *N*-(4′-hydroxycinnamoyl)-3-hydroxyanthranilic acid (YAvn I) and *N*-(3′-4′-dihydroxycinnamoyl)-3-hydroxyanthranilic acid (YAvn II)] have been produced by introducing two plant genes, *4cl-2* from tobacco and *hct* from globe artichoke, in *Saccharomyces cerevisiae* [19] (Figure 2). These yeast-derived AVAs show structural similarity with two major oat AVAs: YAvn I with AVA 2p and YAvn II with AVA 2c, respectively, differing only in the hydroxyl group of the anthranilic moiety [19] (Figure 2).

Lee and colleagues optimized the synthesis of nine AVAs in *Escherichia coli*, using different hydroxycinnamic acids, and providing a new strategy to produce both natural and synthetic AVAs [20], whose biological activities may be explored.

**Figure 3:**
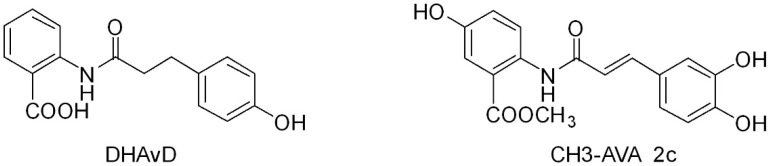
Two examples of AVA synthetic derivatives with significant biological activities: dihydroavenanthramide D and the methylated form of AVA 2c.

Other synthetic derivatives have been produced and evaluated for their biological effects: dihydroavenanthramide D (DHAvD) [21,22,23] (Figure 3), the synthetically methylated form of AVA 2c (CH3-AVA 2c) [24] (Figure 3), and the synthetically methylated form in ring A or B described by Fagerlund et al. [25] (derivatives 3a, 3p, 3c, 3f, and 3s reported in Supplementary Figure S1). The derivatives showed comparable or even higher biological activity than natural AVAs, as described below.

## 3. Pharmacokinetics

The efficacy of phytochemicals is largely dependent on their bioavailability [26]. Understanding the pharmacokinetics of oat phenolic compounds following oral intake of whole oat or oat bran is an important keynote for determining whether AVAs mediate the health benefits of oat.

Few studies evaluated the bioavailability and biotransformation of AVAs in animal models. In one study, BioF1B hamsters were treated by oral gavage with a saline solution containing 0.25 g oat bran phenol-rich powder. The oral dose of each phenolic compound to each animal was: 0.63 µmol 2p; 0.49 µmol 2f; 0.32 µmol *p*-coumaric acid; 0.06 µmol sinapic acid; 0.10 µmol syringic acid; 0.03 µmol p-hydroxybenzoic acid; 0.50 µmol ferulic acid; 0.13 µmol vanillic acid. Blood was collected at 0, 20, 40, 60, 80 and 120 min [27]. Maximum plasma concentration of AVA 2p and 2f and the other phenol acids was reached at 40 min. Although 2p and 2f had the highest concentration in oat bran phenol-rich powder, their apparent relative bioavailability was only 5% of vanillic acid (the least bioavailable phenolic acid) and 1.3% of *p*-coumaric acid (the most bioavailable phenolic acid). Since the concentration of oat phenols was measured only in plasma, it is not possible to exclude the distribution of these compounds in other tissues and/or their biotransformation [27].

Koenig and colleagues investigated the tissue distribution of AVAs in female Sprague-Dawley rats. Rats were treated by oral gavage with a mixture of the three major AVAs (2c, 2f, 2p) at 0.02 g/kg body weight (b.w.) [28]. AVAs concentrations were measured by HPLC in plasma, liver, heart, and gastrocnemius at 0, 1, 2, 4, and 12 h. The overall results showed that AVAs circulated in the blood before reaching the peripheral tissues and were differently taken up by the various organs. All three AVAs were detected mainly as their phase-II conjugated form. In particular, conjugated AVA 2p was the most available in plasma (2.81 ± 0.12, 2.13 ± 0.20, 0.79 ± 0.13, and 1.48 ± 0.68 nmol/L corresponding to 91%, 95%, 91%, and 94% of the total 2p at 1, 2, 4, and 12 h, respectively). The concentration of conjugated AVA 2f was highest in liver and hearth. In the liver, it reached concentrations representing 92%, 87%, 85%, and 90% of total 2f at 1, 2, 4, and 12 h, respectively; in the heart, conjugated AVA 2f was detected at concentrations representing 79%, 100%, and 99% of total 2f at 1, 4, and 12 h, respectively. Conjugated AVA 2c better accumulated in muscle, where it reached concentrations representing 97%, 85%, 80%, and 90% of total 2c at 1, 2, 4, and 12 h, respectively [28].

The pharmacokinetics of AVAs was assessed also in humans. A randomized, placebo-controlled, 3-way crossover trial with a 1-week washout period explored the pharmacokinetics of AVA 2p, 2f, and 2c in healthy older adults [29]. Six subjects (3 males and 3 females; 60.8 ± 3.6 years) drank 360 mL skim milk (placebo) or 360 mL skim milk containing 0.5 or 1 g of an AVA-enriched mixture (AEM) extracted from oat. Concentrations of AVA 2p, 2f and 2c in the AEM were 154, 109 and 111 µmol/g, respectively. The blood draw was done at 15, 30 and 45 min and at 1, 2, 3, 5, 10 h after drink administration. The overall results indicated a different kinetic profile for the three investigated AVAs. Consistent with the findings observed in hamsters and rats, 2p was the most bioavailable AVA in human plasma with the highest maximum plasma concentration after consumption of both 0.5 and 1 g AEM (112.9 and 374.6 nmol/L for 2p, 13.2 and 96.0 nmol/L for 2f, and 41.4 and 89.0 nmol/L for 2c) [29].

The plasma concentration and pharmacokinetic parameters after acute oral intake of oat cookies made of natural oat flour were examined by Zhang and colleagues [30]. 8 males (26.3 ± 3.0 years) and 8 females (24.9 ± 3.4 years) ate three cookies prepared with oat flour at high content of AVAs (H-AVA, 21.61 mg total AVAs, 5.09 mg 2p, 8.67 mg 2f and 7.85 mg 2c) or at low content of AVAs (L-AVA, 3.08 mg total AVAs, 0.57 mg 2p, 0.95 mg 2f and 1.55 mg 2c). Blood samples were collected overall 10 h after ingestion. Authors quantified plasma concentrations of the three AVAs and total AVAs by ultra-high-performance liquid chromatography-mass spectrometry (UPLC-MS). Maximum plasma concentrations for 2p, 2f and 2c were higher in the H-AVA than in the L-AVA group (H-AVA: 2p = 8.39 ± 4.2 ng/mL; 2f = 8.44 ± 2.3 ng/mL; 2c = 4.26 ± 2.0 ng/mL. L-AVA: 2p = 1.98 ± 1.3 ng/mL; 2f = 2.43 ± 1.1 ng/mL; 2c = 1.33 ± 0.7 ng/mL). The maximum plasma concentrations reported in this study for AVAs 2p, 2f and 2c are much lower than those found by Chen et al. [29]. The difference could be attributed to the doses of 2p, 2f and 2c used in the study, which resulted 9-, 4-, and 4-fold higher, respectively, in the paper by Chen et al. [29]. In the H-AVA group, 2f had a longer half-life and a slower elimination rate than 2p and 2c. The pharmacokinetic differences could be ascribed to the high hydrophobicity of 2f (Figure 1), which may slow its elimination. Notably, although the participants ingested a higher dose of AVA 2c than AVA 2p, the plasma concentrations of AVA 2c were much lower than those of AVA 2p. At the same time, AVA 2c showed a lower absorption compared to AVA 2p and AVA 2f, suggesting a different metabolic pathway for AVA 2c [30]. Microbiota has been recognized as a key mediator of pharmacokinetics and biological activity of dietary polyphenols [31,32]. Microbial metabolites of polyphenols can reach human plasma concentrations like those effective in in vitro studies, suggesting their role in the biological effects of polyphenols [33]. AVAs can be metabolized by microbiota [10,34,35,36]. Wang and colleagues found that AVA 2c can be extensively metabolized by mouse and human microbiota to generate several bioactive metabolites (i.e., the reduction product dihydroavenanthramide 2c (DH 2c), the product of hydrolysis, caffeic acid, and the reduction product of caffeic acid). In particular, in mice treated by oral gavage with 200 mg/kg b.w. of AVA 2c, eight metabolites were detected in urine over 24 h: 5-hydroxyanthranilic acid, dihydrocaffeic acid, caffeic acid, dihydroferulic acid, ferulic acid, DH 2c, dihydroavenanthramide 2f (DH 2f), and AVA 2f [34]. Furthermore, in in vitro fecal fermentation experiment, AVA 2c was transformed by the human microbiota into four major metabolites: 5-hydroxyanthranilic acid, dihydrocaffeic acid, caffeic acid, and DH 2c [34]. The multifaceted biotransformation of AVA 2c could explain its lower concentration compared to 2p and 2f observed in the human plasma.

Recently, Schar and colleagues evaluated the urinary excretion of phenolic acid and AVAs in 7 healthy men after intake of 60 g oat bran (7.8 µmol AVAs, 139.2 µmol phenolic acids) by UPLC-MS [37]. The Authors recorded a fast elimination of 30 phenolic compounds within 8 h from the intake. The mean recovery of phenolic compounds detected in the urine was 22.9 ± 5.0% of the ingested phenolic doses. The recovery was lowest for AVA 2p (0.3%). The major metabolites were sulfate- or glycine-conjugated benzoic acids and ferulic acid-*O*-sulfate. These findings indicate that a high percentage of oat phenols are bioavailable as metabolites and that absorption occurs both in the small and large intestine in the 8 h following intake [37].

## 4. Epidemiological Studies

Knowledge about the protective effect of whole-grain intake on cancer development has progressively grown over recent years. However, epidemiological studies on oat and oat products intake are limited.

The large combined Scandinavian HELGA cohort that in 1992–1998 included 120,010 participants aged 30–64 years from three prospective studies (37,231 Norwegian women; 13,294 and 12,431 Swedish woman and men, respectively; 28,875 and 27,178 Danish women and men, respectively) analyzed the intake of different whole-grain types [11]. Information about whole-grain consumption was collected by country-specific food frequency questionnaires (FFQs). Cancer incidence was assessed by national cancer registries. The intake of whole grain oat products significantly reduced all-cause mortality in both sexes [11]. The analysis of whole-grain intake in relation to cause-specific mortality reported a reduction in cancer mortality in individuals who consumed higher amount of breakfast cereals, total whole grain products, oat, and wheat [11]. However, the available information did not point out the effect of oat on the incidence of different types of cancer, as deeply reviewed by Boffetta and colleagues [38].

The incidence of colorectal cancer is related to diet and lifestyle factors [39]. An analysis of the association between whole-grain product intake and risk of colorectal cancer was conducted in HELGA cohort. During a 14-years median follow up, 1123 colorectal cancer cases were diagnosed in the cohort. A lower incidence of colorectal cancer was recorded in individuals who consumed whole grain products. Of note, no significant association was found for the intake of whole grain oat [40]. In line with these findings, oat consumption did not influence the incidence of colorectal cancer cases (n° 1025) observed among the 57,053 participants in Danish Diet, Cancer and Health cohort study over a 13-year period [41].

Regarding other types of gastrointestinal cancers, the HELGA cohort study indicated that high consumption of whole grain products reduced the risk of developing esophageal cancer, although a specific preventive role of oat products was not highlighted [42].

The benefits of whole grain products have been investigated in a population-based case-control study of pancreatic cancer in the San Francisco Bay Area (1995–1999). A 131-item semiquantitative FFQ was administered to 532 cases and 1701 controls. The data set showed that the high daily consumption of whole grain products (two or more servings per day) protected against pancreatic cancer [43]. On the contrary, only a slight positive effect on cancer risk was observed after intake of cooked oatmeal or oat bran [43]. These findings could be imputable to recall bias in reporting past diet habits. Further investigations are needed to understand the effects of oat products on pancreatic cancer risk.

The potential effects of dietary factors on breast cancer risk are of great interest because modifiable risk factors can play an important role in breast cancer prevention [44]. Whole grain products, in addition to fibers, contain several bioactive components that can offer health benefits [45]. Few epidemiological studies evaluated the specific role of oat on breast cancer development. In an analysis of 978 cases of breast cancer diagnosed among 24,418 women in the Danish Diet, Cancer and Health cohort study over a 9.6-year follow up time, no association was found between the intake of oatmeal and the risk of breast cancer [44].

The population-based AGES-Reykjavik cohort study investigated the association between consumption of various food categories in adolescence and midlife and the risk of breast cancer among 3326 women (age 77.0 ± 6.0 years). At the beginning of the study, the participants filled a FFQ on diet in adolescence, in midlife and at the entry of the study (late life). During a mean 8.8-year follow up, 97 women were diagnosed with breast cancer. Interestingly, a continuous elevated intake of oatmeal in adolescence and midlife was associated with a reduced risk to develop the disease [46].

Prostate cancer is the second most frequent cancer in men [47]. A growing body of evidence reports that diet may be implicated in the etiology of prostate cancer. The relationship between intake of specific whole-grain products, including oatmeal, and prostate cancer was investigated among 26,691 men (50–64 years) who participated in the Danish diet, Cancer and Health prospective cohort study. During a median 12.4-year follow up, 1081 prostate cancer cases were identified. Overall, no association between oatmeal intake and prostate cancer risk was observed as well as with total intake of whole-grain products [48]. Additionally, 2268 men (age: 67–86 years) included in the AGES-Reykjavik cohort study reported their food habits for early, middle and current life using validated FFQ. The data set indicated that the intake of oat both in adolescence or midlife did not exert a protective effect on prostate cancer risk [49].

Prospective epidemiological studies presented in this review are limited and mainly involve a study cohort including Scandinavian people which traditionally eat oat products. The set of data indicates a protective effect of whole grains intake, including oat products, on the overall mortality and cancer risk, however the influence of oat on the incidence of different types of cancer cannot be assessed.

An open problem of epidemiological studies is the dietary assessment. The FFQs used are validated and common standardization guidelines are employed. Despite this, measured errors are often an issue with self-reported data. Furthermore, in prospective cohort study, diet is assessed only at baseline and does not reflect the changes during the relatively long follow-up period. In addition, participants may not correctly report other confounding factors (e.g., smoke and alcohol consumption) associated with cancer risk, which can bias the results.

Human studies focusing on oat dietary intake and health benefits are warranted to understand the role of oat food in the prevention of cancer and eventually support the formulation of dietary recommendations and guidelines encouraging an efficacious consumption of whole oat grains.

## 5. Cancer Chemopreventive Activity of AVAs

Antioxidant activity is crucial in preventing or relieving cancer by reducing the oxidative damage to cellular components induced by reactive oxygen species (ROS) [50]. Phenols are well-known antioxidants playing a key role in cancer chemoprevention [51].

Oat contains a wide range of molecules that act as antioxidants, including AVAs, which share structure similarity with polyphenols, but have antioxidant capacity 10–30 times greater than that of other oat’s phenolic compounds, such as caffeic acid or vanillin [52,53]. Several studies explored the antioxidant activity of AVAs (Table 1).

One the most widely used method to evaluate antioxidant ability of dietary components is the oxygen radical absorption capacity (ORAC) assay [52]. ORAC assay combines inhibition time and inhibition percentage of reactive species into a single quantity. Whilst the exact relationship between the ORAC value of a compound and its health benefit has not been fully established, compounds with higher ORAC scores have in general greater antioxidant ability [60].

Although AVAs represent a major portion of the phenolics in oat, Chu and colleagues [52] demonstrated the absence of a direct correlation between AVAs content and ORAC score in seven different common cultivars of oat (Table 1). Since the authors analyzed the whole oat groats, it is possible that others plant constituents including tocopherols, sterols and phytic acids play a role in the final antioxidant capacity of oat [52].

On the other hand, Chen and colleagues [54] found a correlation between AVAs content and ORAC values in flour from nine Chinese oat varieties and bran from four oat varieties (Table 1). In most cases, phenolic contents and antioxidant capacities of oat brans were higher than in whole oat. Additionally, Antonini and colleagues [55] determined AVAs content and ORAC values of two different Italian oat cultivars, Donata and Flavia. Donata raw grains had 2.8-fold higher AVAs content and an ORAC value 30% higher than Flavia. Interestingly, this study offers some important information: (1) the soil type is crucial for the preservation of AVAs content and oat biological activity (Table 1); (2) oat processing significantly modifies AVAs content and ORAC values. Indeed, the content of AVAs and the ORAC values are higher in the raw grain than in the dehulled groat or flour for both Donata and Flavia cultivars. Other studies quantified the free radical scavenging capacities of the three most abundant AVAs isolated from oat, meaning AVA 2c, 2f and 2p. Scarpa and colleagues compared the antioxidant activity, evaluated through the ORAC assay, of two oat dried fractions: one characterized by the presence of AVA 2f at 95% (named P3) and one characterized by the presence of an AVAs mixture, meaning AVA 2c (37%), 2f (8%) and 2p (35%) for the 80% of the total weight of the fraction (named P4) [56]. Minor AVA forms characterized the remaining 20% of the P4 fraction. The higher ORAC value of P4 compared to P3 (19,079 vs. 6547, respectively) (Table 1) together with the quantified amount of the different AVAs in P3 and P4 allows hypothesizing the key role of AVA 2c in the greater antioxidant activity of P4 [56]. This hypothesis is supported by the results of a study assessing the specific antioxidant capacity of AVA 2c, 2f or 2p against peroxyl radicals (ORAC assay), hydroxyl radicals (HORAC assay), superoxide anion [superoxide anion absorption capacity (SORAC) assay], singlet oxygen [singlet oxygen absorption capacity (SOAC) assay], and peroxynitrite [peroxynitrite absorption capacity (NORAC) assay] (Table 1). AVA 2c was endowed with the best in vitro antioxidant capacity [17].

The different antioxidant activity among AVAs is imputable to their different chemical structures. Within this framework, Fagerlund and colleagues deeply investigated the relationships between AVAs’ structures and the corresponding radical scavenging activity, measured by 2,2-dyphenyl-1-picryldrazyl (DPPH) assay [25]. Unsubstituted AVAs (1a, Supplementary Figure S1) are very poor antioxidants. The introduction of one hydroxyl group does not lead to a significant increase in the antioxidant activity (1p and 2a vs. 1a, Supplementary Figure S1). The antioxidant power increases by introducing a second hydroxyl group or a methoxy group in the *ortho* position in the ring of the cinnamic part compared to the derivative having one hydroxyl group (i.e., 2c and 2s vs. 2p, Supplementary Figure S1). Of note, the introduction of a methoxy group in the ring of the anthranilic part leads to a major antioxidant activity compared to its introduction in the other ring (3p vs. 2f) (Supplementary Figure S1). Lee-Manion and colleagues [57] performed a similar study taking in consideration only AVAs of the series 1 and 2 (Supplementary Figure S1). Authors found out that the most active AVA was 2c followed by 2p and 2s. This is in contrast with the paper by Fagerlund et al. [25] reporting that 2c is less or slightly less active than 2s, and that 2p is basically inactive. However, this incongruence may be due to variations in the reaction conditions, such as AVAs’ concentrations and tested times. Further data obtained using different in vitro assays (Table 1) confirmed AVA 2c as the main antioxidant of oat [50,57,59]. AVA 2c showed an antioxidant activity similar to that of butylated hydroxytoluene (BHT), used as positive control [50]; both AVA 2c and 2f had higher antioxidant activity than 6-hydroxy-2,5,7,8-tetramethylchromane-2-carboxylic acid (Trolox^®^) [50] (Table 1). The highest in vitro antioxidant activity of AVA 2c may be due to the *ortho*-hydroxyl group on the cinnamic acid moiety [17,25,57].

To evaluate the ability of oat phytochemicals to penetrate cells and scavenge intracellular free radicals, Scarpa and colleagues [56] performed some experiments on the cellular antioxidant activity (CAA) of the previously described oat fractions (P3 and P4) on colorectal and liver tumor cells (Caco2 and HepG2, respectively) (Table 1). Cells were incubated for 24 h with the two different preparations at the lowest active concentrations. A statistically significant antiproliferative effect was recorded on the two tested cell lines. Then, ROS were generated using H_2_O_2_. P4 had the strongest CAA in both Caco2 and HepG2 cell lines. Furthermore, both P3 and P4 significantly reduced intracellular ROS levels. The intracellular antioxidant activity of AVA 2f was confirmed in a human hepatoma cell line (Hep3B), where it prevented the intracellular H_2_O_2_-mediated formation of ROS [58] (Table 1). An intracellular scavenger activity was demonstrated also for the synthetic derivative DHAvD: DHAvD treatment of human dermal fibroblasts significantly reduced UVB-induced ROS formation [21] (Table 1).

Chen and colleagues [54] showed a lack of correlation between AVAs’ content and CAA values for flour from nine Chinese oat varieties and bran from four oat varieties in HepG2 cells (Table 1). The conflicting evidence on the correlation between AVAs content and CAA values can be explained by a different uptake of the tested preparations in colorectal and liver cell lines. The physical-chemical characteristics of oat flour, rich in fiber content, can obstacle AVAs bioavailability compared to P3 and P4, which are AVAs enriched extracts. Molecular size, polarity and solubility of the preparation also contribute to a different uptake at cellular level [54].

Studies on isolated AVAs help understanding their antioxidant mechanisms. A very recent study explored the antioxidant activity of AVA 2c in normal human skin fibroblasts exposed to extracellular oxidative stress [59]. AVA 2c reduced the levels of intracellular free radicals and contrasted the up-regulation of antioxidant gene transcripts induced by oxidative stress. In particular, treatment with 100–200 µM AVA 2c for 48 h followed by 1 h H_2_O_2_ treatment prevented the up-regulation of antioxidant enzymes, including glutathione synthetase (GSS), heme oxygenase 1 (HMOX1), superoxide dismutase 1 (SOD1), glutathione peroxidase 1 (GPX) and catalase (CAT), induced by H_2_O_2_ [59].

Together with their scavenger activity against H_2_O_2_-induced oxidative stress, AVAs activate the antioxidant defensive pathways. In human fibroblasts, AVA 2c up-regulated heme oxygenase-inducible form at both gene (HMOX1) and protein (HO-1) level in a concentration-dependent fashion. In mammals, heme oxygenase is a crucial antioxidant pathway that degrades heme, a pro-oxidant molecule, and produces biliverdin, Fe^2+^, and CO. The activation of HO-1-protection pathway is regulated by the nuclear factor erythroid-related factor 2 (Nrf2) [59]. Nrf2 plays a pivotal role in contrasting exogenous or endogenous oxidative insults and controlling cellular oxidative status [61]. Nrf2 is sequestered in the cytoplasm by the actin-binding protein Kelch-like ECH associating protein 1 (Keap1). When the redox-sensitive cysteines of Keap-1 are oxidized, Nrf2 migrates into the nucleus, where it binds to the antioxidant response element of phase II antioxidant enzymes and boosts gene expression [62]. AVA 2c induced the expression of HO-1, a phase II antioxidant enzyme, through an increased DNA-binding activity of Nrf2 in fibroblast monolayer. These findings were also described by Fu and colleagues [62] on human renal proximal tubule cells (Hk-2), a cellular model widely used to test the nephroprotective effects of phytochemicals via Nrf2 pathway. AVA 2c, 2f and 2p induced the expression of HO-1.

AVAs present in their structure an α,β-unsaturated moiety, a well-known Michael acceptor, characteristic of molecules such as curcumin and caffeic acid phenethyl ester that are, like AVAs, HO-1 inducers. Unsurprisingly, AVAs analogues, in which the double bond of the α,β-unsaturated system has been reduced, do not show any ability to increase the expression of HO-1, indicating that this system is critical for their antioxidant potential [62].

Mechanistic studies revealed that HO-1 expression induced by AVAs was mediated via Nrf2 translocation and ROS. HO-1 activation could be mediated by mitogen-activated protein kinase (MAPK) signaling pathway or phosphoinositide 3-kinase (PI3K) pathway [63], but induction of HO-1 by AVAs was independent of both of them. In fact, inhibitors of MAPK or PI3K did not alter the activation of HO-1 pathway caused by AVAs [62]. Treatment with N-acetylcysteine (NAC), a well-known ROS scavenger, abolished the AVAs-induced HO-1 activation. On the whole, the collective body of evidence indicate that the key role in HO-1 activation is played by ROS and not by PI3K or MAPK [62].

**Figure 4:**
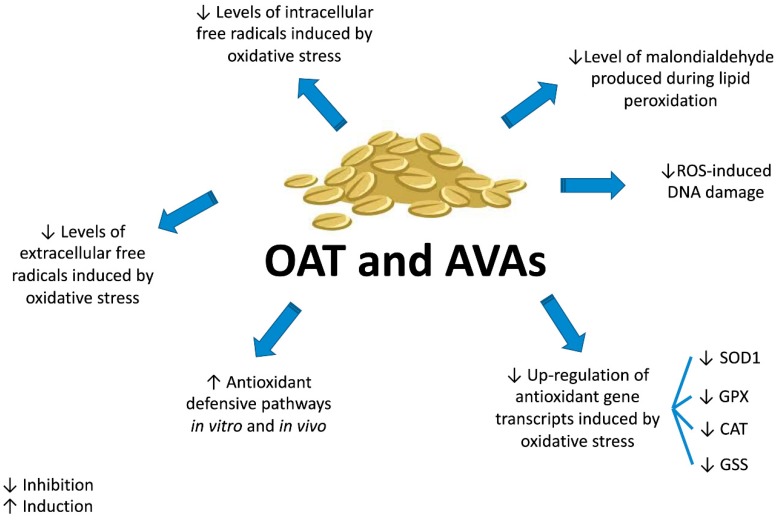
Antioxidant activity of AVAs from oat.

ROS-induced oxidative stress can increase DNA damage [57,64]. The comet assay, based on single cell gel electrophoresis, was used to assess the potential protective effect of AVAs against the DNA damage induced by H_2_O_2_ on human colon adenocarcinoma cells (HT29). AVA 2c had the highest activity against H_2_O_2_-induced DNA damage, with an EC_50_ (concentration decreasing H_2_O_2_-induced DNA damage by 50%) of 0.9 ± 0.4 µM. The genoprotective activity was higher than that of ascorbic acid (EC_50_: 1.6 ± 0.0 µM), a well-known antioxidant [57]. Furthermore, AVA 2c was evaluated for its ability to protect cells from H_2_O_2_-induced DNA damage using the H2A.X phosphorylation assay. Pre-treatment of primary dermal fibroblasts with 100 and 200 µM AVA 2c decreased the H2A.X phosphorylation induced by free radicals, further supporting the cytoprotective effects of AVA 2c [59]. Taken together, these in vitro results suggest for AVAs (1) a scavenger activity at both intracellular and extracellular level, (2) an increase in cell antioxidant defenses, and (3) a decrease in ROS-induced DNA damage (Figure 4).

Of note, the genoprotective ability of AVAs is not restricted to ROS-induced DNA damage. An AVA-enriched extract of oats administered daily to male Sprague-Dawley rats at a dose of 20 mg/kg b.w. by gastric tube for 6 weeks reversed the DNA damage caused by TiO_2_ nanoparticles [65].

In vivo studies confirm the antioxidant and protective activity of AVAs. Rats fed for 50 days with AVAs (0.10 g/kg b.w.) exhibited greater SOD activity in the vastus lateralis muscle, liver and kidney and higher GPX activity in the heart and vastus lateralis muscle compared to untreated animals [66]. Furthermore, Ren and colleagues [67] investigated the effects of an AVAs-rich extract (ARE) from oat bran in D-galactose stressed mice. Mice were treated with ARE 0.25, 0.50, and 1.00 g/kg b.w. per day by intragastric gavage for 2 weeks after D-galactose injection. ARE restored the antioxidant defenses compromised by the D-galactose administration, up-regulating the expression of SOD and GPX. Besides, ARE diminished the level of malondialdehyde (MDA) [67], a toxic compound generated during lipid peroxidation initiated by ROS [68]. On the whole, these findings suggest that the up-regulation of SOD and GPX enforces the in vivo antioxidant pathways and can be involved in the in vivo defense against oxidative stress damage.

## 6. Anticancer Effects of AVAs

AVAs are able to modulate several events, such as apoptosis, cell proliferation, metastatization, specifically involved in all stages of cancer development, as proved by many mechanistic studies evaluating their anticancer activity in various cancer cell lines (Table 2) and in animal models. 

### 6.1. In Vitro Studies

#### 6.1.1. Antiproliferative Effects and Induction of Apoptosis

**Figure 5:**
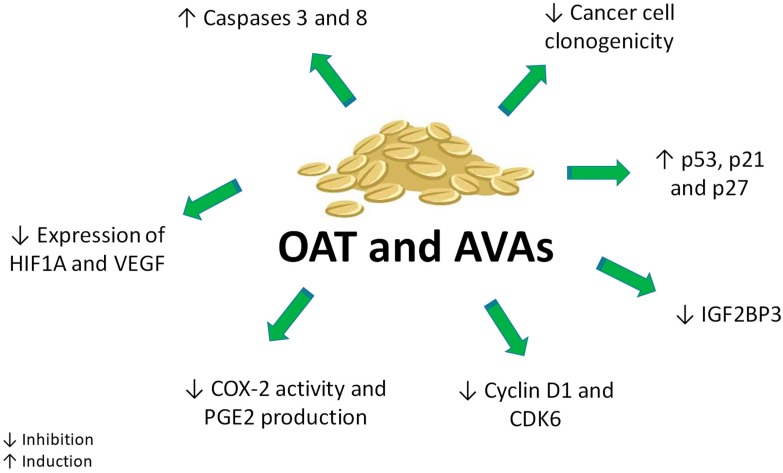
Antiproliferative and pro-apoptotic activity of AVAs from oat.

Inflammation plays a well-known role in tumor development. Accordingly, 25% of cancers are related with chronic inflammation. Colonic carcinogenesis offers an important example of the association between chronic inflammation and cancer development: chronic ulcerating colitis and Crohn’s disease boost the risk of colorectal cancer up to tenfold [72]. Many studies report the association between prostaglandin (PG)-E2 signaling and cancer development in the gastrointestinal tract [73]. Moreover, cyclooxygenase-2 (COX-2), catalyzing PG biosynthesis and expressed in cells of tumor stroma such as macrophages, is involved in colon carcinogenesis and overexpressed in tumor tissues when compared with normal tissues [73,74]. Guo and collaborators explored the antiproliferative effect of an AVAs-enriched extract (AvExO), AVA 2c, and the synthetically prepared methylated form of AVA 2c (CH3-AVA 2c) on colon cancer cell lines (HT29, LS174T, HCT116) [24]. AvExO, AVA 2c and its methylated form induced an antiproliferative effect, independently of COX-2 expression and PGE2 production (Table 2). CH3-AVA 2c exhibited the highest activity. This effect may be attributed to the presence of the methyl ester group in its structure, which could boost its lipophilicity, favoring its passage through the cell membrane. In the same study, the Authors reported that AvExO did not affect the expression of COX-2 protein, but downregulated its activity and the production of PGE2 in mouse primary macrophages [24]. Taken together, those results suggest that AVAs exert their effect on colon cancer cells through mechanisms dependent and independent of inflammation (Figure 5).

Other studies explored the antiproliferative activity of different oat varieties [54] or fractions [56] (Figure 5). Seven different oat varieties and four oat brans, all characterized by a different phenolic content (Table 1), evoked antiproliferative but not cytotoxic effects up to 250 mg/mL in human hepatocellular carcinoma cells (Table 2). This means that the antiproliferative activity is independent from cell death, but is related to the variability of polyphenol content of oat [54]. Scarpa and colleagues evaluated the antiproliferative activity of the aforementioned fractions P3 (containing AVA 2f at 95%) and P4 [containing AVA 2c (37%), 2f (8%) and 2p (35%)] in Caco-2 and HepG2 cell lines. P4 was more active in cell-growth inhibition compared to P3 in both tested cell lines (Table 2). This result could be explained by the highest cell permeability and bioavailability characterizing AVA 2c and 2p when compared to those of AVA 2f, the main component of the P3 fraction [29,56]. The same fractions were tested for their ability to induce apoptosis [56].

Apoptosis is triggered by cascade-cascade system, whose regulation is tiny controlled by cysteine aspartyl-specific proteases, also known as caspases [75]. Caspases are divided into three subfamily: apoptosis activators, apoptosis executioners, inflammatory mediators [75]. There are two apoptotic pathways: the intrinsic or mitochondrial pathway, where there is the release of different apoptogenic factors such as cytochrome *c*, apoptosis-inducing factor, etc. from the mitochondrial intermembrane space and the involvement of the initiator caspase-2 and -9, which in turn activate the effector caspase-3; the extrinsic pathway, triggered by the death receptors of the tumor necrosis factor (TNF) receptor superfamily such as CD95 (APO-1/Fas) or TNF-related apoptosis-inducing ligand receptors, which involves the activation of the initiator caspase-8 and effector caspase-3 [75].

The effects of AVAs on caspases 8, 9 and 3 were evaluated in different cancer cell lines (Figure 5). Caspase-8 and caspase-3 activity was significantly increased in Caco-2 cells by both P3 and P4, tested at 120 µM, whereas in HepG2 cells only P4 induced a significant activation of caspase-3 and caspase-8. On the other hand, neither P3 nor P4 activated caspase-9, thus excluding the involvement of the intrinsic pathway in their pro-apoptotic effects [56]. The ability of AVA 2f to activate the extrinsic apoptotic pathway via caspase-8 was also demonstrated in Hep3B hepatocarcinoma cell line [58] (Table 2). Those data were supported by the lack of modulation of survivin mRNA by AVA 2f in HepG2, Caco2 and Hep3B cell lines [56,58]. Survivin is codified by BIRC5 (Baculoviral inhibitor of apoptosis repeat-containing 5), a member of apoptosis protein family inhibitors, whose expression suppresses apoptosis via caspase-9 inhibition [76]. Moreover, Scarpa and colleagues explored the influence of AVAs on the modulation of two pro-survival genes in cancer cells: the vascular-endothelial growth factor (VEGF) and the hypoxia-inducible factor 1-alpha (HIF1A) [56]. Both VEGF and HIF1A allow cancer cells coping with oxidative stress and unbalanced redox status arising from the rapid growth and scarcity of oxygen and nutrients of advanced tumors. Cancer cells can contrast oxidative stress by the up-regulation of HIF1A and VEGF, which provides more oxygen to cancer cells [77]. P4 mixture significantly down-regulated the expression of VEGF and HIF1A on both Caco2 and HepG2 cancer cells. Thus, the blockage of adaptive responses mediated by VEGF/HIF1A in cancer cells represents a convergent anti-survival effect evoked by AVAs.

The cytotoxic activity of AVAs was also evaluated on breast cancer cell lines. In MDA-MB-231 triple negative (estrogen receptor, progesterone receptor, human epidermal growth factor receptor 2) mammary tumor cells, AVA 2c, 2p and 2f induced cytotoxic effects in a time- and concentration-dependent manner (Table 2). However, the cytotoxic effect of AVA 2p and 2f was weaker than that of AVA 2c. Those results suggest that AVA 2c acts through a different mechanism of action: AVA 2c was the only one able to massively increase the sub-G1 population, indicative of an apoptotic mechanism, as confirmed via propidium iodide (PI)/annexin-V staining and caspase-3/7 activation [69].

The antiproliferative activity of AVA 2c, AVA 2p and the yeast-derived recombinant AVAs (i.e., YAvn I and II) was investigated on HT29 cells through the colony formation assay [18]. All natural and yeast-derived AVAs showed comparable activity in inhibiting HT29 clonogenicity (Table 2). Additionally, these compounds inhibited cell-cycle progression by the up-regulation of cell-cycle inhibitor proteins including p53, p21 and p27 (Table 2) (Figure 5). The antiproliferative effect of AVA 2f and YAvn I and II was also recorded on cervix adenocarcinoma cells (HeLa) [19], where it was related to the inhibition of cyclin D1 at non cytotoxic concentrations (Table 2).

Oat bran contains many AVAs in form of glycosides [16]. Wu and colleagues observed that both the glycosylated (AVA 2c-O-glc) and non-glycosylated form of AVA 2c exerted similar growth inhibitory activity on two colon cancer cell lines, HT29 and HCT116. This means that glycosylation did not affect the antiproliferative effects of AVAs (Table 2).

AVAs are transformed in the gastrointestinal tract in biologically active metabolites by human microbiota [34,35]. Inhibitory effects were reported not only for AVA 2c and AVA 2f, but also for their metabolic products generated by the intestinal microbiota on colon cancer cells’ growth. Interestingly, the bioactive metabolites of AVA 2c and 2f (DH 2c and DH 2f, respectively) had higher antiproliferative activity than their precursors (Table 2). AVA 2c and DH 2c were also tested for their ability to induce apoptosis [34]: only DH 2c significantly triggered apoptosis (Table 2). These results show that biotransformation not only retains AVAs’ biological activities, but also that AVAs’ dihydro-metabolites have greater anticancer effects compared to their precursors.

#### 6.1.2. Induction of Senescence

The induction of cellular senescence is another potential mechanism postulated for the tumor-suppressive activity of AVAs [70]. Senescence refers to the irreversible cell-cycle arrest occurring when cells are under stress conditions, such as oxidative stress, telomere shortening, genomic damages, and metabolism dysfunction. The morphological and biochemical hallmarks of senescence include enlarged cellular size, increase in β-galactosidase activity, telomere shortening, and enhanced expression of p53 and p21 [78].

AVA 2p triggered cellular senescence in colon cancer cells (HCT116 and HCT8), as indicated by specific morphological and biochemical features including cellular enlarged size and increased β-galactosidase activity, together with H2A.X positive staining and cell-cycle arrest in G1 phase [70]. Furthermore, AVA 2p significantly increased the expression of miR-129-3p. MiRNAs are non-coding RNA able to silence RNA and regulate gene expression at post-transcriptional level. A growing body of evidence identifies miRNAs as key players in cancer [79]. The increased expression of miR-129-3p induced by AVA 2p markedly repressed Pirh2 (p53-induced RING-H2), an E3 ubiquitin ligase [70]. Pirh2 participates in an autoregulatory feedback loop that controls p53 expression [80]. The increased expression of miR-129-3p induced by AVA 2p silenced Pirh2, which in turn increases the expression of p53 and its downstream target p21, leading to senescence (Table 2). In addition, Fu and colleagues [70] reported that AVA 2p treatment down-regulated other two targets of the tumor suppressor miR-129-3p: IGF2BP3 (insulin like growth factor 2 mRNA binding protein 3) and CDK6 (cyclin-dependent kinase 6), both important for G1-phase progression and G1/S-transition [81]. The down-regulation of IGF2BP3 and CDK6 contributes to AVA 2p-induced cell-cycle arrest (Figure 5) and senescence.

Taken together, those results indicate that AVA 2p induces senescence in colon cancer cells via the activation of miR-129-3p/Pirh2/p53 signaling pathway. Thus, AVA 2p seems to be a valuable candidate for the auxiliary activation of tiny mechanisms such as miRNAs regulation to suppress colon cancer growth.

#### 6.1.3. Inhibition of Epithelial-Mesenchymal Transition and Metastatization

Cancer metastatization is the major cause of cancer-related death [82]. The capacity of anticancer drugs to target and prevent this process is fundamental to improve clinical outcomes. During the epithelial-mesenchymal transition (EMT), epithelial cells acquire a mesenchymal state with metastatic capacities [83], enhanced mobility, invasion and resistance to apoptosis [84]. The main hallmark of EMT is the loss of epithelial surface markers, with changes in cell-cell adhesion, cell polarity and differentiation properties. These events are mainly caused by the modification of proteins such as E-cadherin, which regulates epithelial cell adhesion, and vimentin, a mesenchymal protein [83].

Finetti and colleagues [18] demonstrated on human colorectal cancer cells (HT29, WiDr) that the major natural AVAs (2p and 2c) and the yeast-derived recombinant AVAs (YAvn I and II) were equally effective in the inhibition of tumor cell growth and survival (Table 2). However, YAvn I and II resulted more effective in blocking EMT and reducing tumor cell migration. The expression of E-cadherin was measured to understand the difference in EMT reduction between natural and yeast-derived AVAs. YAvn I and II resulted more effective in down-regulating E-cadherin compared to natural AVAs. The modulation of E-cadherin expression by yeast AVAs occurred through the down-regulation of the zinc finger protein Snail1 and the lymphoid-enhancing factor-1 (LEF-1) (Table 2), two transcriptional factors that facilitate the transition from the epithelial to the mesenchymal state via E-cadherin suppression [85]. Tumor cell invasion and metastasis require degradation of the extracellular matrix (ECM), which acts as a biochemical and mechanical barrier to cancer cells’ movement. Matrix metalloproteinases (MMPs) are responsible for ECM degradation and thus are crucial for cancer progression [86]. Among MMPs, MMP-9 expression is related to metastatization in many tumors, including breast cancer [87]. A variety of stimuli, such as cytokines and treatment with the tumor promoter 12-O-tetradecanoylphorbol-13-acetate (TPA), are able to activate transcription factors like nuclear factor-kappa B (NF-κB) and activator protein-1 (AP-1) leading to MMP-9 expression [88]. AP-1 activation strongly depends on MAPK signaling pathway [89].

An AVA-enriched extract, AVA 2c and its methylated form, and the synthetic analogue DHAvD inhibited the cytokine-induced activation of NF-κB [23,90]. DHAvD was also tested for its ability to inhibit breast cancer cells’ invasion mediated by MMP-9 expression [22], whose gene promoter contains binding sites for NF-κB [91]. In MCF-7 cells, DHAvD was able to inhibit TPA-induced MMP-9 expression through the block of both MAPK and NF-κB signaling (Table 2).

Wingless (Wnt) proteins and their downstream effectors regulate many processes, including tumor initiation, tumor growth, cell senescence, cell death, cell differentiation, and metastasis [92]. In Wnt pathway, β-catenin acts as a central element. Many human cancers are characterized by an aberrant β-catenin signaling [92], which is involved in the modulation of the metastatic potential of tumor cells. Accordingly, the attenuation of its constitutive activation is an attractive target for cancer prevention and therapy [71]. Wang and colleagues [71] demonstrated the pro-apoptotic effects of AVA 2p in human cervical cancer cells, mediated by the abrogation of aberrant β-catenin signaling (Table 2). AVA 2p decreased β-catenin in the cytosol, diminished its nuclear accumulation, and reduced the subsequent transcriptional activation of Wnt target genes such as *c-Myc*, an oncogene that is overexpressed in a wide variety of human cancers and drives metabolic alterations of cancer cells [71]. An open question is whether AVA 2p directly targets these molecular events or acts as an upstream regulator.

### 6.2. In Vivo Studies

Despite many in vitro studies exploring AVAs’ anticancer potential, scanty in vivo studies investigated the anticancer activity of AVAs. A very recent study [93] analyzed the antineoplastic activity of AVA 2c methyl ester on female Swiss Albino mice intraperitoneally injected with Ehrlich ascites carcinoma cells, which generate aggressive murine mammary adenocarcinoma. Treatment of mice with 50 ng/kg/day of AVA 2c methyl ester, administered by oral gavage for two weeks, reduced tumor volume and growth [93]. More in details, levels of serum tumor markers such as alpha-fetoprotein (AFP) and carcinoembryonic antigen (CEA) were increased in the group of mice inoculated with Ehrlich carcinoma cells. Both these markers were significantly reduced in the AVA-treated group [93]. In addition, AVA contrasted the decrease in CAT, GSH and SOD levels and the increase in MDA levels observed in tumor tissues [93]. In mice with tumor, an increase in TNF-α plasma levels was also observed. The increase in this pleiotropic cytokine could be due to a rinse in macrophage oxidative stress [94]. AVA treatment significantly decreased TNF-α levels in tumor sections [93], confirming the scavenger activity of AVAs, widely described in vitro. Finally, the study analyzed the different expression of genes involved in the apoptotic pathway. AVA administration increased the expression of p53 and decreased the expression of Bcl-2 in mice suffering from tumor. A decrease in the proliferating cell nuclear antigen (PCNA) was also observed, indicating the inhibition of tumor cell proliferation by AVA treatment [95]. Furthermore, AVA inhibited NF-κB-dependent intracellular signaling [93], which regulates the expression of many genes involved in tumor cell invasion and angiogenesis, such as MMPs, COX-2, chemokines and inflammatory cytokines [96].

Although the study reported above analyzed different biomarkers to support the in vivo anticancer activity of AVAs, its main limitation is the insufficient characterization of administered AVAs. Nevertheless, an interesting data emerging from the study is the absence of toxic effects in the group of mice treated only with AVAs, leading to assume their safe profile after 14 days of repeated administration.

Fu and colleagues [70] recently tested the in vivo ability of AVA 2p to contrast the colorectal cancer development using a well-recognized animal model of colitis-associated carcinoma. In particular, male C57BL/6J mice were treated with a single intraperitoneal injection of azoxymethane (AOM), a genotoxic carcinogen, and dextran sodium sulfate (DSS), a non-genotoxic carcinogen, in drinking water for 7 consecutive days. The AOM/DSS mice were then fed with 30 mg/mL/day of AVA 2p for 14 days. AVA 2p reduced tumor incidence and tumor diameter of macroscopic polyps if compared with animals treated only with AOM/DSS [70]. Due to the interesting anticancer properties observed for AVA 2p, in the same study the Authors performed a biosafety evaluation of AVA 2p. In particular, they performed a microscopic analysis of different organs including heart, liver, spleen, and kidney in mice fed with AVA 2p. No morphological changes were recorded [70].

## 7. Selectivity of AVAs towards Cancer Cells

Most of anticancer drugs are afflicted by frequent and severe toxic effects, mainly imputable to their inability to selectively target cancer cells. Bearing in mind the importance of selectivity towards cancer cells for promising anticancer compounds, the available data on AVAs selectivity are reported below. Guo and colleagues explored the in vitro selectivity of AVAs in Caco-2 cells [24]. Caco-2 cells are an ideal model for comparing the anticancer effect of a compound on Caco-2 proliferating cells vs. the effect on differentiated Caco-2 cells, which acquire the characteristic phenotype of normal non-transformed colonic epithelial cells [24]. AVAs (AVA 2c, CH3-AVA 2c) and an extract described above (AvExO) tested in the range 0–120 µM (AVA 2c and AvExO) or 0–40 µM (CH3-AVA 2c) inhibited cell proliferation of Caco-2 cells, whereas at the same concentrations they had no effect on differentiated Caco-2 cells [24], indicating a selective antiproliferative effect for cancer cells. The lack of antiproliferative activity of AVAs was also observed on normal human keratinocytes (NCTC 2544) [56], which were treated with the P3 and P4 fraction reported above in the range 0–120 µM. Moreover, AVA 2p at 7.5 and 15 µM induced senescence in colorectal cancer cells, but not in the non-transformed counterpart (i.e., colon epithelial cells) [70]. AVA 2c and 2p and the two yeast-derived recombinant YAvn I and II tested in the range 0–200 µM decreased cell viability in colorectal cancer cells (HT29 and WiDr), but not in normal colon fibroblasts (CCD-18Co) [18].

On the whole, these results on isolated AVAs and extracts univocally disclosed the selectivity of AVAs towards tumor cells, encouraging further studies to exploit AVAs’ anticancer potential.

## 8. Conclusions

**Figure 6:**
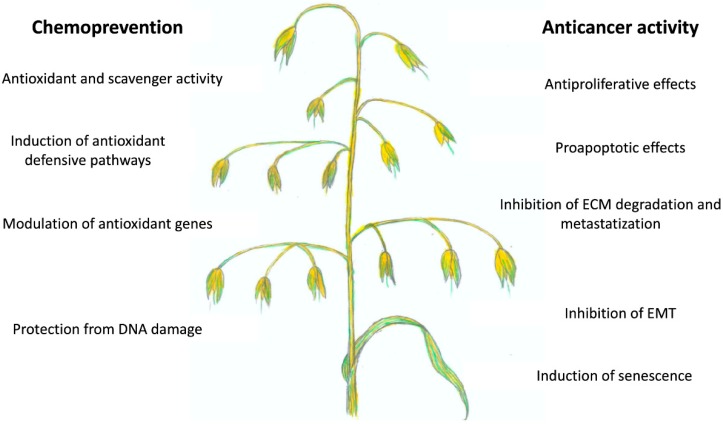
Chemopreventive and anticancer activity of AVAs from oat.

For more than 4000 years, *Avena sativa* has been recognized as a food and its traditional use has been recorded since the 12th century. Recent in vitro and in vivo investigations highlighted the potential therapeutic effects of oat preparations and its signature compounds AVAs in the oncological field. In particular, AVAs are able to modulate many events involved in cancer development (Figure 6).

However, only a couple of clinical trials are available for AVAs. A randomized, placebo-controlled, double-blind pilot study analyzed the effects of AVA-enriched bran on inflammation biomarkers demonstrating its ability in reducing the levels of the pro-inflammatory cytokine vascular cell adhesion molecule-1 in older, overweight or obese adults [97]. No effects were recorded on the levels of serum amyloid A-1 [97], an acute phase reactant in inflammation. A further clinical trial explored a different aspect of oat, not related to its antitumor properties: its ability to protect against the rash caused by different antitumor drugs including cetuximab, erlotinib, panitumumab, and sorafenib. A colloidal oatmeal lotion controlled the rash and allowed continuation of the antineoplastic therapy [98].

A critical topic to better predict the potential health benefit of AVAs is the characterization of their bioavailability and distribution in humans. It is well known that compounds exhibiting promising pharmacological properties on in vitro assays, often show an unfavorable pharmacokinetic profile. Up to date the pharmacokinetic profile of AVAs has not yet been exhaustively investigated. In vivo studies highlighted that the three major AVAs (2p, 2f, 2c) are poorly absorbed in the gastrointestinal tract and AVA 2c shows the lowest bioavailability. Human studies confirmed this limited bioavailability and indicated pharmacokinetic differences among AVAs related to their chemical structures. However, these studies present limitations in the experimental design, including the low number of enrolled individuals and the variability of AVAs’ source.

Last but not least, very limited data are available on the safety profile of oat preparations and AVAs. In an acute toxicity study, aortic endothelial cells were treated with an AVA-enriched mixture at doses up to 40 µg/mL. No cytotoxic effects were recorded [98]. Furthermore, in a sub-chronic toxicity study, an AVA-enriched extract of oats was administered daily to male Sprague-Dawley rats at a dose of 20 mg/kg b.w. by gastric tube for 6 weeks. No alterations in hematological parameters, testosterone levels, and liver, brain, lung, kidney, heart and testes of treated animals were recorded [65]. No data are available on chronic and reproductive toxicity of oat preparations and AVAs.

The prediction of genotoxicity is an important toxicological issue for the definition of the risk/benefit profile of a compound. Genotoxicity leads to mutations in various cells and mutations play a critical role in cancer and a wide variety of different diseases. A couple of study examined the genotoxicity of AVAs. Lee-Manion and colleagues [57] treated colon adenocarcinoma cell line HT-29 with AVAs 2p, 2c, 2f or 2s (0.5 or 2.5 µM). No DNA damage was observed using the comet genotoxicity assay. Those in vitro results were confirmed by a recent in vivo study. An AVA-enriched extract of oats was administered daily to male Sprague-Dawley rats at a dose of 20 mg/kg b.w. by gastric tube for 6 weeks. No DNA damage was observed by comet assay in the liver tissue of treated animals [65]. Bearing in mind that DNA damage can be expressed in the cell as gene mutation, structural chromosomal aberration, and genomic changes, the available studies do not allow to draw a conclusion about the genotoxic potential of AVAs.

In conclusion, the overall findings discussed in this review highlight the interesting properties of oat AVAs, potentially for both preventive and therapeutic interventions. Those properties rely on their chemical structure that provides antioxidant activity and modulates cellular and molecular events involved in multiple stages of cancer development. However, clinical trials and safety studies with oat preparations or AVAs are not yet sufficient to support their efficacy in patients at cancer risk; and this aspect can be considered as an emerging science.

## Figures and Tables

**Table 1:** Intracellular and extracellular in vitro antioxidant activity of oat cultivars or avenanthramides (AVAs).

Product Tested	Assay	Quantification of Antioxidant Activity	Phenolic/AVAs Content	Ref.
7 cultivars of whole groats	ORAC	11–28 µmol Trolox equivalents/g	Phenolic content (mg/g gallic acid equivalent): 0.57–0.94	[52]
			Total AVAs content (mg/kg): 9.44–163.36	
Flour or bran from different Chinese varieties	ORAC CAA	18.02–25.62 µmol Trolox equivalents/g	Total phenolic content (mg/g dry weight): 52.82–81.20	[54]
		25.31–33.38 µmol of quercetin equivalents/100 g	Total AVAs content (mg/g dry weight): 5.01–214.26	
Two varieties cultivated in different soil types	ORAC	Free phenols (FPs): 46.3–195.3 µmol Trolox equivalents/g dry weight	Free phenols (FPs) content: 1.27–1.99 mg/g dry weight	[55]
			Bound phenols (BPs) content: 2.02–2.71 mg/g dry weight	
			AVAs content: 68.5–417.4 µg/g dry weight	
P3 fraction P4 fraction	ORACCAA DCFH-DA ^a^	P3: 6547 µmol Trolox equivalents/g; P4: 19,079 µmol Trolox equivalents/g	AVAs average percentages present in each fraction: P3: 95% AVA 2f; P4: mixture of 37% AVA 2c, 8% 2f and 35% 2p	[56]
		CAA: P4 > P3 in both HepG2 and Caco2		
		DCFH-DA: P4 > P3		
AVA 2c, 2f, 2p	ORAC	17,860–36,818 µmol Trolox equivalents/g		[17]
	HORAC	16,240–19,915 µmol Trolox equivalents/g		
	NORAC	1044–3038 µmol Trolox equivalents/g		
	SORAC	8334–47,729 µmol Trolox equivalents/g		
	SOAC	5059–20,089 µmol Trolox equivalents/g		
AVA 2c, 2f, 2p	DPPH assay β-carotene assay	EC_50_^c^ (µmol): AVA2c: 0.074; AVA 2f: 0.105; AVA 2p: 0.198	Phenolic content (gallic acid equivalent, mol/mol): AVA 2c: 1.89; AVA 2f: 1.09; AVA 2p: 0.93	[50]
		EC_50_ (µmol): AVA2c: 0.0029; AVA 2f: 0.018; AVA 2p: 0.074		
AVA 2c, 2p, 2f	DPPH assay FRAP ^b^ assay	AVA 2c: 6.1 µmol; AVA 2p: 5.7 µmol; AVA 2f: 3.3 µmol		[57]
		EC_1_^d^, (µmol/L): AVA 2c: 275; AVA 2p: 343; AVA 2f: 422		
AVA 2f	DCFH-DA	Reduction of H_2_O_2_-induced ROS		[58]
DHAvD	DCFH-DA	Reduction of UVB-induced ROS		[21]
AVA 2c	Mitotracker Orange	Reduction of H_2_O_2_-induced oxidative stress		[59]

^a^ DCFH-DA: dichloro-dihydro-fluorescein diacetate; ^b^ FRAP: ferric reducing antioxidant power; ^c^ EC_50_: half maximal effective concentration; ^d^ EC_1_: equivalent concentration 1, concentration of compound having ferric-2,4,6-tripyridyl-s-triazine reducing ability equivalent to 1 mmol/L FeSO_4_·7H_2_O.

**Table 2:** In vitro anticancer activity of oat cultivars or AVAs.

Product Tested	Cell Line	Assay (Treatment Time)	Range of Concentrations, EC_50_^a^ or IC_50_^b^	Anticancer Effects	Ref.
AvExO, AVA 2c, CH3-AVA 2c	Caco-2, HT29, LS174, HCT116	MTT ^c^ assay (48 h)	AvExO (40–160 µM); AVA 2c (40–160 µM); CH3-AVA 2c (1–80 µM)	Antiproliferative effects	[24]
Flour or bran from different Chinese varieties	HepG2	MTT assay (72 h)	EC_50_: 167.31–233.42 mg/mL	Antiproliferative effects	[54]
P3 fraction P4 fraction	Caco-2, HepG2	Sulforhodamine B assay and caspase activation (48 h)	IC_50_ Caco2 (µM): P3: 126.5 ± 12.5; P4: 114.6 ± 5.5 IC_50_ HepG2 (µM): P3: 182.7 ± 18.1; P4: 39.9 ± 4.1	Apoptosis via activation of caspases 8 and 3 Down-regulation of VEGF and HIF1A.	[56]
AVA 2f	Hep3B	Sulforhodamine B assay and caspase activation (48 h)	IC_50_: 240 ± 10 µM	Apoptosis via activation of caspase-8	[58]
AVA 2c, DH 2c, AVA 2f, DH 2f	HCT116	MTT assay (24 h)	IC_50_ (µM): AVA 2c: 363; DH 2c: 158; AVA 2f: >400; DH 2f: 257	Antiproliferative effects Apoptosis induction by DH 2c	[34]
AVA 2c, AVA 2c-O-glc	HCT116, HT29	MTT assay (48 h)	IC_50_ HCT116 (µM): AVA 2c: 319.7; AVA 2c-O-glc: 301.1 IC_50_ HT29 (µM): AVA 2c: 326.8; AVA 2c-O-glc: 389.9	Antiproliferative effects	[16]
AVA 2c, AVA 2f, AVA 2p	MDA-MB-231	MTT assay; caspase activation (48–96 h)	50–400 µM	Antiproliferative effects Apoptosis activation via caspase-3/7 (AVA 2c)	[69]
AVA 2c, AVA 2p, YAvn I, YAvn II	HT29, WiDr	Colony formation assay; MTT assay; gene and protein expression (72 h)	50–200 µM	Antiproliferative effects Up-regulation of p53, p21, p27 Inhibition of EMT and tumor migration Down-regulation of E-cadherin by YAvn I and II via Snail1 and LEF-1 down-regulation	[18]
AVA 2f, YAvn I, YAvn II	Hela	MTT assay; cyclin D1 expression (24 h)	25–150 µM	No cytotoxic effect up to 150 µM Decrease in cyclin D1 expression	[19]
AVA 2p	HCT116, HCT8	Gene and protein expression (3, 5, 7 days)	7.5 or 15 µM	Senescence induction via the activation of miR-129–3p/Pirh2/p53 signaling pathway Down-regulation of IGF2BP3 and CDK6	[70]
DHAvD	MCF7	MTT assay (24 h)	1–20 µM	MMP-9 down-regulationNF-κB inhibitionInhibition of metastasis and invasion	[22]
AVA 2p, AVA 2f	HeLa	MTS ^d^ assay (48 h)	20–160 µM	Antiproliferative and pro-apoptotic effects of AVA 2p through abrogation of aberrant β-catenin signalingc-Myc down-regulation	[71]

^a^ EC_50_ half maximal effective concentration; ^b^ IC_50_ half maximal inhibitory concentration; ^c^ (3-[4,5-dimethylthiazol-2-yl]-2,5 diphenyl tetrazolium bromide); ^d^ (3-[4,5-dimethylthiazol-2-yl]-5-[3-carboxymethoxyphenyl]-2-[4-sulfophenyl]-2H-tetrazolium).

## Supplementary Materials

The following are available online at https://www.mdpi.com/1422-0067/20/18/4536/s1.

## Abbreviations

AEM	AVA-enriched mixture
AFP	alpha-fetoprotein
AOM/DSS	azoxymethane/dextran sodium sulfate
AP-1	activator protein-1
ARE	AVAs-rich extract
AVA 2c-O-Glc	AVA 2c glycosylated
AVAs	Avenanthramides
AvExO	AVAs enriched extract
b.w.	body weight
BHT	butylated hydroxytoluene
CAA	cellular antioxidant activity
CAT	Catalase
CDK6	cyclin-dependent kinase 6
CEA	carcinoembryonic antigen
COX-2	cyclooxygenase-2
DCFH-DA	dichloro-dihydro-fluorescein diacetate
DH 2c	dihydroavenanthramide 2c
DH 2f	dihydroavenanthramide 2f
DHAvD	dihydroavenanthramide D
DPPH	2,2-dyphenyl-1-picryldrazyl
ECM	extracellular matrix
EMT	epithelial-mesenchymal transition
FFQs	food frequency questionnaires
FRAP	ferric reducing antioxidant power
GPX	glutathione peroxidase 1
GSH	Glutathione
GSS	glutathione synthetase
H-AVA	high amount of AVAs
HMOX1	heme oxygenase 1
HO-1	heme oxygenase 1
HORAC	hydroxyl radical absorption capacity
IGF2BP3	insulin like growth factor 2 mRNA binding protein 3
Keap1	Kelch-like ECH associating protein 1
L-AVA	low amount of AVAs
LEF-1	lymphoid-enhancing factor-1
MAPK	mitogen-activated protein kinase
MDA	Malondialdehyde
MEK1	mitogen-activated protein kinase kinase
MiRNA	microRNA
MMP	matrix metalloproteinase
MTS	(3-[4,5-dimethylthiazol-2-yl]-5-[3-carboxymethoxyphenyl]-2-[4-sulfophenyl]-2H-tetrazolium)
MTT	(3-[4,5-dimethylthiazol-2-yl]-2,5 diphenyl tetrazolium bromide)
NAC	N-acetylcysteine
NF-κB	nuclear factor-kappa B
NORAC	peroxynitrite absorption capacity
Nrf2	nuclear factor erythroid related factor 2
ORAC	oxygen radical absorption capacity
PCNA	proliferating cell nuclear antigen
PGE2	prostaglandin-E2
PI3K	phosphoinositide 3-kinase
Pirh2	E3 ubiquitin ligase p53-induced RING-H2
ROS	reactive oxygen species
SOAC	singlet oxygen absorption capacity
SOD	superoxide dismutase
SORAC	superoxide anion absorption capacity
TNF	tumor necrosis factor
TPA	12-O-tetradecanoylphorbol-13-acetate
Trolox	6-hydroxy-2,5,7,8-tetramethylchromane-2-carboxylic acid
UPLC – MS	ultra-high-performance liquid chromatography – mass spectrometry
YAvn I	*N*-(4′-hydroxycinnamoyl)-3-hydroxyanthranilic acid
YAvn II	*N*-(3′-4′-dihydroxycinnamoyl)-3-hydroxyanthranilic acid
