# Supplementary figures and images for: Overview of the Anticancer Profile of Avenanthramides from Oat
Source: Int J Mol Sci. 2019 Sep 13;20(18):4536. doi: 10.3390/ijms20184536 (PMC6770293; doi:10.3390/ijms20184536)

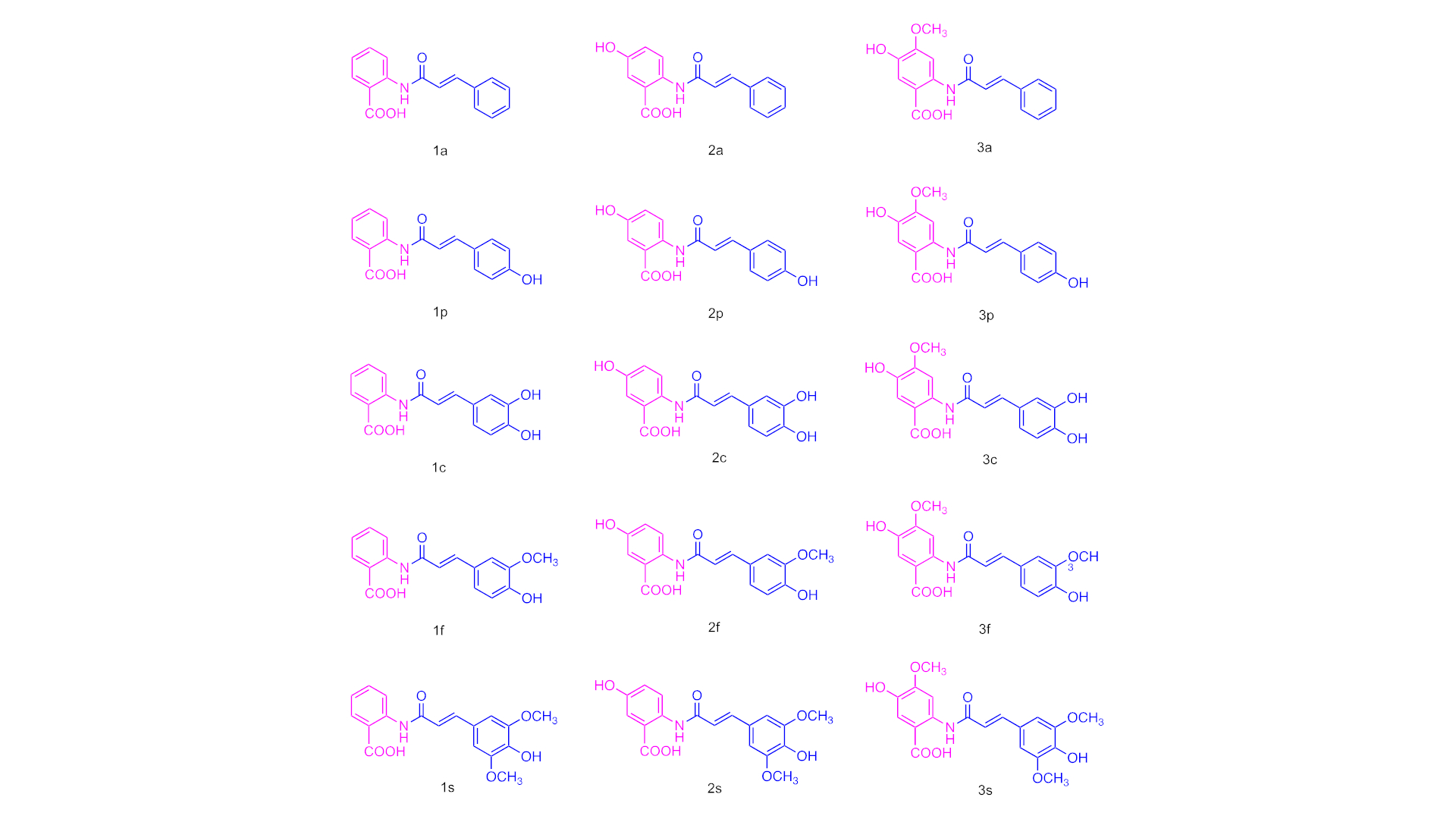

Supplement: Supplementary file 1 [file ijms-20-04536-s001.jpg]
